# Cardiovascular–Cognitive Associations During Physiological Challenges in Relapsing-Remitting Multiple Sclerosis

**DOI:** 10.3390/diseases14090343

**Published:** 2026-09-17

**Authors:** Juan M. Racosta, Jorge Arocha-Perez, Da Hee Seo, Samin Ayromlou, Patricia M. Riccio

**Affiliations:** 1 Department of Clinical Neurological Sciences, University Hospital, London Health Sciences Centre, London, ON N6A 5A5, Canada; jorge.arochaperez@lhsc.on.ca (J.A.-P.); dahee.seo@lhsc.on.ca (D.H.S.); samin.ayromlou@lhsc.on.ca (S.A.); patricia.riccio@lhsc.on.ca (P.M.R.); 2 Schulich School of Medicine & Dentistry, Western University, London, ON N6A 5C1, Canada

**Keywords:** multiple sclerosis, information processing speed, symbol digit modalities test, autonomic dysfunction, exercise

## Abstract

Background: Cognitive impairment, particularly reduced information processing speed (IPS), is a clinically meaningful manifestation of multiple sclerosis (MS). The Symbol Digit Modalities Test (SDMT) is a sensitive, validated measure of IPS widely used in MS research. While vascular and hemodynamic factors are increasingly recognized as contributors to cognitive performance in MS, their relationship with IPS across distinct physiologic challenges remains unexplored. Objective: This pilot study examined IPS and cardiovascular responses in ten people with MS (PwMS), all of whom had relapsing-remitting MS (RRMS), and ten age- and sex-matched healthy participants during four acute physiologic challenges: head-up tilt, head-down tilt, isometric handgrip, and aerobic cycling. Methods: Beat-to-beat blood pressure and heart rate were continuously recorded; IPS was assessed using the oral SDMT. Results: Baseline SDMT scores were lower in PwMS than in healthy participants (*p* < 0.05). A between-group effect was observed for cycling-related SDMT change (*r* = 0.49, 95% CI 0.05–0.76; *p* = 0.03). Despite comparable training impulse (TRIMP)-standardized workloads, systolic blood pressure during cycling was higher in PwMS (*p* < 0.05), and the cycling-related systolic blood pressure response showed a between-group effect of *r* = 0.46 (95% CI 0.02–0.75; *p* = 0.04). In healthy participants, baseline SDMT performance was correlated with resting heart rate and blood pressure, and cycling-related SDMT change was inversely correlated with the blood pressure response. Conclusions: These exploratory findings suggest possible differences in the relationship between cardiovascular measures and IPS during acute physiologic challenges, particularly aerobic exercise. However, the small sample size and wide confidence intervals preclude firm conclusions regarding an exercise-specific effect or differences in cardiovascular–cognitive coupling.

## 1. Introduction

Multiple sclerosis (MS) is a chronic immune-mediated disease of the central nervous system characterized by inflammatory demyelination, neuroaxonal injury, and neurodegeneration. Although MS has traditionally been defined by motor, sensory, visual, and gait-related disability, cognitive impairment is now recognized as one of its most clinically meaningful manifestations. Cognitive dysfunction may occur early in the disease course, may be present even in patients with mild physical disability, and can substantially affect employment, social functioning, treatment adherence, and quality of life. Among the cognitive domains affected in MS, information processing speed (IPS) is one of the most consistently impaired and is considered a core cognitive phenotype of the disease [1,2,3].

The Symbol Digit Modalities Test (SDMT) is widely used to assess IPS in people with MS (PwMS) because it is brief, sensitive, reproducible, and clinically feasible. The SDMT captures the rapid integration of visual scanning, attention, working speed, and response generation, and it has been recommended as a valid cognitive performance outcome measure for MS clinical research. Compared with longer neuropsychological batteries, the SDMT offers a practical method for repeated assessment of cognitive performance across experimental conditions, including conditions that may transiently alter physiological state [1,4].

The mechanisms underlying IPS impairment in MS are multifactorial. Conventional explanations emphasize lesion burden, white matter disconnection, gray matter atrophy, thalamic injury, and altered functional network efficiency [1,5]. However, increasing evidence suggests that vascular and hemodynamic factors may also contribute to cognitive performance in MS [6,7,8]. Among these factors, reduced arterial compliance and impaired cerebrovascular reactivity have been proposed as potential contributors to neurovascular uncoupling and cognitive slowing in MS [9].

Exercise is particularly relevant because it acutely alters heart rate, blood pressure, vascular tone, and cerebral hemodynamics, all of which may interact with cognitive performance. Prior studies examining acute exercise and cognition in MS have produced mixed findings. A study reported that acute treadmill walking improved reaction time on a modified flanker task in people with relapsing-remitting MS (RRMS) compared with quiet rest [10]. In contrast, another study found that acute aerobic exercise transiently increased mean arterial pressure and carotid artery stiffness and reduced middle cerebral artery conductance in people with RRMS but did not result in exercise-specific improvements in modified flanker task performance relative to a seated control condition [11]. However, to our knowledge, no studies have examined IPS and hemodynamic responses together across different forms of acute physiologic stress in MS, including orthostatic stress, anti-orthostatic positioning, isometric handgrip, and aerobic cycling. The present pilot study was designed to examine IPS and cardiovascular responses in people with RRMS compared with age- and sex-matched healthy participants (HP).

## 2. Materials and Methods

### 2.1. Participants

The study included 10 PwMS, all of whom had RRMS, and 10 age- and sex-matched HP. All participants were between 18 and 59 years of age. Participants aged 60 years or older were excluded to minimize potential confounding effects of age-related changes in IPS, autonomic regulation, arterial stiffness, and cerebrovascular function, thereby facilitating the evaluation of MS-related physiological responses [12,13]. Before testing, HP underwent a neurological examination by a neurologist (JMR), which confirmed normal neurological findings and no clinical evidence of autonomic or hemodynamic dysfunction. Participants were excluded if they met any of the following criteria: (i) pregnancy or lactation; (ii) clinically significant coronary artery disease; (iii) failure of other organ systems or systemic illness that could affect autonomic or hemodynamic function or the ability to cooperate with study procedures; or (iv) evidence of another medical cause of cognitive impairment, such as attention-deficit/hyperactivity disorder or traumatic brain injury. The inclusion criterion for PwMS was a clinical diagnosis of RRMS according to the 2017 revised McDonald criteria [14], confirmed by a neurologist (JMR). PwMS were recruited from the MS Clinic at University Hospital in London, Ontario. Ethical approval was obtained from the Health Sciences Research Ethics Board at Western University (HSREB 107108).

### 2.2. Study Protocol

**Figure 1:**
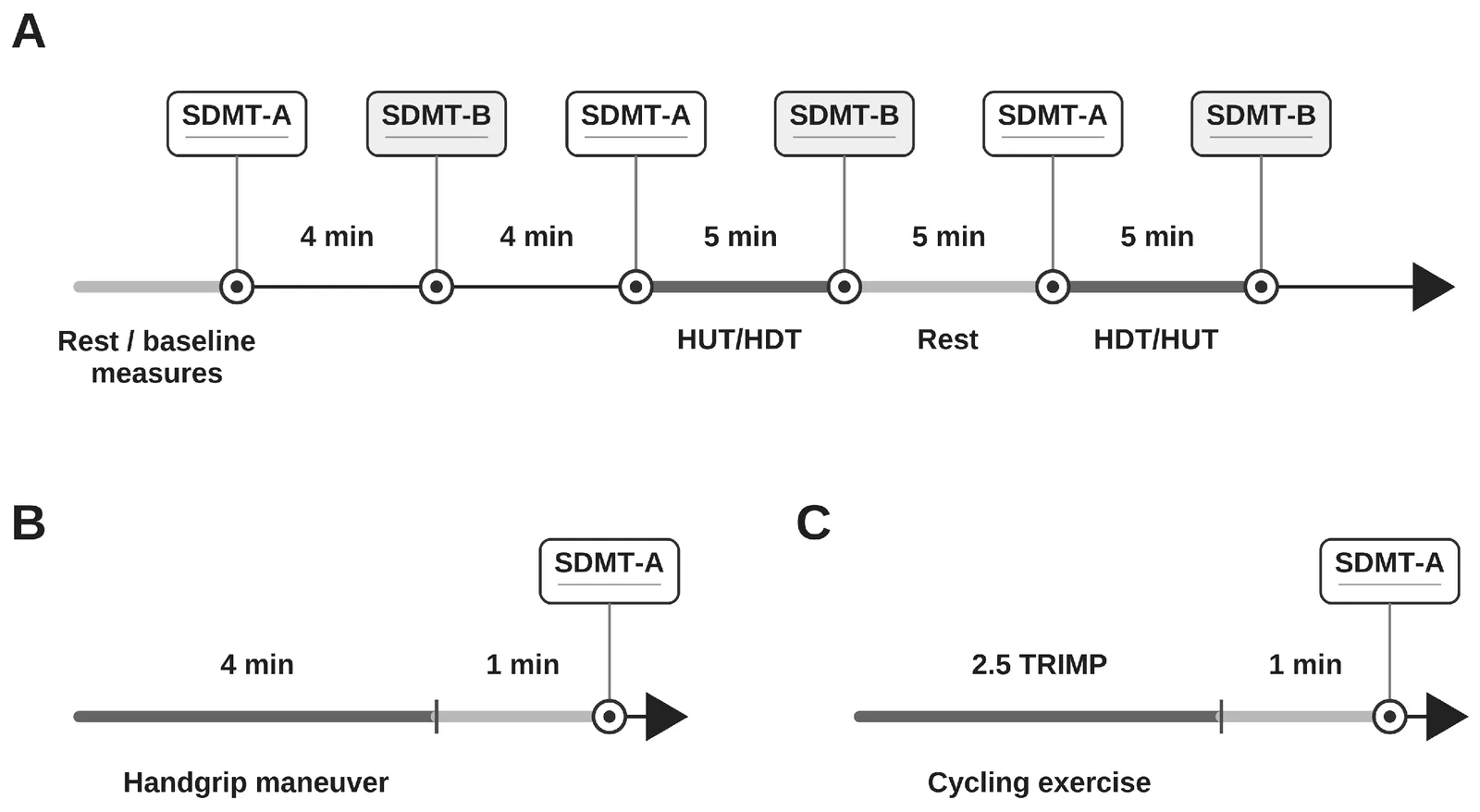
Study protocol completed by each participant. (**A**) Tilt protocol, including baseline supine rest, repeated SDMT administrations, and alternating head-up tilt and head-down tilt conditions; (**B**) Isometric handgrip protocol, consisting of a 4 min handgrip maneuver followed by SDMT administration after a 1 min recovery period; (**C**) Cycling protocol, individualized to achieve 2.5 training impulse (TRIMP) units, followed by SDMT administration after a 1 min recovery period. HDT, head-down tilt; HUT, head-up tilt; SDMT, Symbol Digit Modalities Test; TRIMP, training impulse; min, minutes.

The study protocol and timing of SDMT administrations are illustrated in Figure 1. Two alternate versions of the SDMT were used to reduce repeated exposure to the same test while maintaining consistency within paired comparisons. SDMT-a was administered at SDMT-1, SDMT-3, SDMT-5, SDMT-7, and SDMT-8, whereas SDMT-b was administered at SDMT-2, SDMT-4, and SDMT-6. The order of the initial orthostatic challenge, head-up tilt versus head-down tilt, was alternated across consecutive individuals to distinguish the effect of performing the SDMT in a non-supine position from the effect of sympathetic activation secondary to the orthostatic challenge. The same SDMT version, SDMT-b, was administered during both head-up tilt and head-down tilt to allow comparison of the same test version between the two tilt conditions. Likewise, SDMT-a was used before and after the handgrip and cycling interventions. This approach prioritized consistency within paired comparisons rather than equalizing the total number of administrations of each SDMT version across the protocol. The first three SDMT assessments during the orthostatic protocol, SDMT-1, SDMT-2, and SDMT-3, were separated by 4 min intervals to provide consistent spacing between repeated cognitive assessments, reduce immediate carryover from the preceding 90 s test, maintain a stable resting condition before the orthostatic challenges, and avoid unnecessarily prolonging the protocol. Subsequent SDMT assessments were administered after each 5 min tilt or recovery period, using SDMT-4, SDMT-5, and SDMT-6, respectively; therefore, these intervals reflected the prespecified duration of the orthostatic challenges and recovery periods rather than a validated SDMT washout interval. SDMT-7 was administered 1 min after completion of the 4 min handgrip maneuver. After handgrip, participants rested until heart rate and blood pressure returned to baseline before beginning the cycling protocol; therefore, the interval between SDMT-7 and SDMT-8 varied according to the recovery period and individualized cycling duration. SDMT-8 was administered 1 min after completion of cycling exercise to assess cognitive performance in close temporal proximity to the physiological response elicited by exercise.

Before head-up tilt and head-down tilt, all participants rested quietly in the supine position for at least 15 min while baseline measures were recorded. Following baseline, participants were passively tilted to 70° from the horizontal position for 5 min during the head-up tilt condition. The tilt angle was selected in accordance with published recommendations for cardiovascular autonomic testing [15,16,17]. Head-down tilt was performed at −10° from the horizontal position for the same duration as head-up tilt to facilitate within-subject comparisons between postural interventions. After the initial tilt maneuver (head-up tilt or head-down tilt), participants returned to the supine position for an additional 5 min resting recovery period before undergoing the alternate tilt. For participants who completed head-up tilt first, head-down tilt was performed second, and vice versa.

In the supine position, participants completed a submaximal isometric handgrip exercise using a Jamar hand dynamometer with the non-dominant hand. Before completing the exercise, participants performed a maximal voluntary contraction to calibrate the dynamometer to 25% of maximal voluntary contraction. Participants then rested quietly in the supine position for 1 min while baseline measures were recorded. Isometric handgrip consisted of a sustained isometric contraction for 4 min at 25% maximal voluntary contraction, based on commonly used isometric exercise protocols [18]. Following the completion of the handgrip exercise, participants rested in the supine position until heart rate and blood pressure returned to baseline values before beginning the cycling protocol. Exercise duration during cycling was individualized according to the training impulse (TRIMP) calculation (see Section 2.4).

### 2.3. Blood Pressure and Heart Rate Assessment

All participants completed the Autonomic Symptom Profile (ASP) questionnaire. Beat-to-beat blood pressure and heart rate were measured continuously during rest in the supine position and during all maneuvers using a non-invasive continuous hemodynamic monitoring system (BMEYE Nexfin, Amsterdam, The Netherlands) and an electrocardiography device (Model 3000 Cardiac Trigger Monitor, IVY Biomedical Systems, Inc., Branford, CT, USA) with electrocardiography electrodes (Ambu^®^ Blue Sensor SP, Glen Burnie, MD, USA), respectively. All recordings were made using WR TestWorks™ software, version 3.3.1 (WR Medical Electronics Co., Stillwater, MN, USA).

Average resting heart rate (rHR) and resting blood pressure (rBP) were recorded during baseline in the supine position. During tilt, postural change in heart rate (ΔHR) was calculated by subtracting rHR from the maximal heart rate recorded between minutes 2 and 5 of each head-up tilt and head-down tilt condition (ΔHR-HUT and ΔHR-HDT). Postural change in blood pressure (ΔBP-HUT and ΔBP-HDT) was calculated similarly.

During isometric handgrip exercise, changes in heart rate and blood pressure (ΔHR-HG and ΔBP-HG) were calculated by subtracting average rHR and rBP from maximal heart rate and blood pressure achieved between minutes 2 and 4 of handgrip. Perceived exertion was assessed using the Borg Rating of Perceived Exertion Scale (RPE) [19].

### 2.4. Cycling Exercise

**Figure 2:**
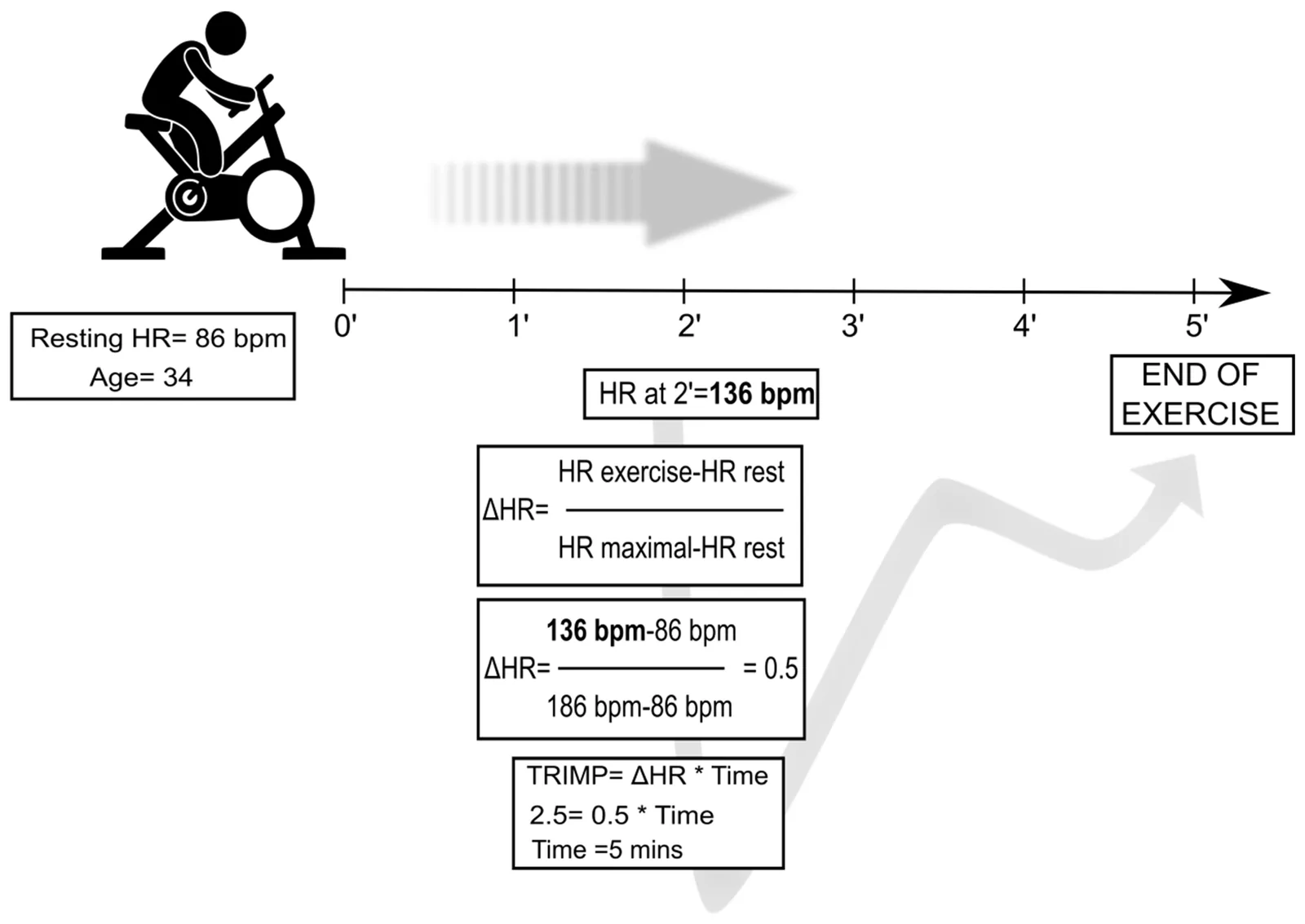
TRIMP calculation. Baseline heart rate and heart rate measured after two minutes of exercise were used to calculate the duration of exercise equivalent to 2.5 TRIMP units for the specific participant. In this case, exercise is stopped after 5 min. bpm: beats per minute; ΔHR, delta heart rate; HR, heart rate; TRIMP, training impulse; min, minutes; * represents a multiplication sign.

Aerobic cycling sessions were performed on a stationary bike at a steady pace of 60 revolutions per minute and a resistance of 1 kg. Training workload, defined as the exercise dose completed by participants, was obtained using the TRIMP method, which integrates exercise duration and average heart rate (beats per minute) [20]. A target workload of 2.5 TRIMP units was selected in this study because it corresponds to a short exercise duration (ranging from 5 to 12 min) in HP (Figure 2). To obtain a workload of 2.5 TRIMP, delta heart rate (ΔHR) was used as the main exercise variable and was calculated as follows: ΔHR = (HR_exercise_ − rHR)/(HR_maximal_ − rHR). The duration of cycling session in both HP and PwMS was then obtained by dividing 2.5 TRIMP by ΔHR. Heart rate and blood pressure were continuously recorded during a 2 min baseline period before the onset of exercise. Blood pressure measurements were obtained with participants’ arms positioned at heart level. Heart rate and blood pressure changes during cycling session were calculated by subtracting the average rHR and rBP before cycling session from maximal heart rate and blood pressure during cycling session (ΔHR-BC and ΔHR-BC).

### 2.5. Information Processing Speed Assessment

IPS was assessed using the oral version of the SDMT [21]. Participants were instructed to verbally match each symbol to its corresponding number as quickly as possible within 90 s using the reference key printed at the top of the stimulus page. Two SDMT versions were used: SDMT-a (SDMT-1, SDMT-3, SDMT-5, SDMT-7, and SDMT-8) and SDMT-b (SDMT-2, SDMT-4, and SDMT-6) (Figure 1). After receiving instructions, each participant completed a 10-symbol matching practice session. Total raw scores were recorded for each SDMT administration, and test-to-test changes between the consecutive assessments were calculated. All tests were administered by the same investigator (JMR).

### 2.6. Outcomes and Statistical Analysis

The main IPS outcomes were: (i) baseline IPS before any intervention (SDMT-1 raw score); and (ii) changes in SDMT raw scores after each intervention (head-up tilt and head-down tilt = SDMT-2 to SDMT-4 and SDMT-4 to SDMT-6, handgrip = SDMT-5 to SDMT-7, and cycling session = SDMT-7 to SDMT-8). Raw scores from all SDMT administrations were analyzed as secondary outcomes to assess whether the primary findings based on change scores were consistent across repeated testing.

The main hemodynamic outcomes were: (i) rHR and rBP; (ii) ΔHR-HUT and ΔBP-HUT; (iii) ΔHR-HG and ΔBP-HG; and (iv) ΔHR-BC and ΔBP-BC. Secondary variables included demographic data, ASP scores, tympanic temperature change, and clinical variables, including Expanded Disability Status Scale (EDSS) score, mean time since last relapse, annualized relapse rate, and age at disease onset.

The statistical analysis was divided into three categories. First, baseline SDMT raw scores and improvement in consecutive SDMT scores were compared between groups. SDMT raw scores and improvement in consecutive SDMT scores within each individual were also compared to assess whether baseline IPS was associated with subsequent improvement. Baseline SDMT raw scores were compared with demographic data and clinical variables.

Second, mean resting heart rate and blood pressure, as well as mean heart rate and blood pressure changes after each intervention (head tilt, isometric handgrip, and cycling session), were compared between groups. Individual heart rate and blood pressure changes after the three interventions were also compared with each other within both groups to assess the ability of each physiological challenge to elicit different cardiovascular responses. All hemodynamic measures were compared with demographic data and clinical variables.

Third, IPS measures and their corresponding hemodynamic measures were examined: association between baseline SDMT raw scores with resting heart rate and blood pressure were assessed; SDMT change after isometric handgrip was compared with heart rate and blood pressure changes during isometric handgrip; and SDMT change after cycling session was compared with heart rate and blood pressure changes during cycling session.

Given the relatively small sample size, all statistical analyses were performed using nonparametric methods. Correlations were assessed using Spearman’s rank correlation coefficient and approximate 95% confidence intervals. Group comparisons were performed using the Mann–Whitney U test, with effect size expressed as *r* = Z/√N, where Z is the standardized Mann–Whitney statistic and N is the total sample size, and 95% confidence intervals for r. All tests were two-tailed. Because the analyses were exploratory and were not adjusted for multiple comparisons, *p* values were interpreted together with effect-size magnitude and confidence-interval precision rather than as a binary threshold for significance. Results are expressed as mean or median ± standard deviation (SD) or range, as appropriate. All statistical analyses were performed using SPSS Statistics, version 22.0 (IBM Corp., Armonk, NY, USA).

## 3. Results

### 3.1. Demographic and Patient Characteristics

Ten HP and ten PwMS completed the study. Eighty percent of participants were female in both groups. The mean age was 39.4 ± 9.6 years for HP and 34.4 ± 5.8 years for PwMS (*p* > 0.05). Mean EDSS in the PwMS group was 2.0 ± 1.1, annualized relapse rate over the previous 5 years was 0.2 ± 0.1, mean time from last relapse was 37 months (range = 8 to 90 months), and age at disease onset was 30.3 ± 7.2 years. At the time of testing, all 10 PwMS were receiving a current disease-modifying therapy (DMT), including dimethyl fumarate (40%), intramuscular or subcutaneous interferon beta-1a (40%), teriflunomide (10%), or natalizumab (10%). All participants had received their current DMT for more than 1 year, with a mean treatment duration of 4.6 ± 4.2 years. Nine participants had received only their current DMT, whereas the participant receiving natalizumab had previously been treated with glatiramer acetate. One PwMS was also taking several non-DMT medications that could potentially influence hemodynamic or autonomic function, including ramipril, nadolol, silodosin, amitriptyline, and fampridine. One healthy participant was taking amitriptyline. When considered clinically safe, these participants withheld the relevant non-DMT medications for approximately 24 h before testing to reduce acute post-dose effects; this interval was not intended to represent a complete pharmacologic washout.

The mean cycling session workload was standardized between two groups, averaging 2.53 ± 0.25 TRIMP units in HP and 2.56 ± 0.37 TRIMP units in PwMS. Exercise duration was 7.4 ± 1.8 min in HP and 6.7 ± 2.8 min in PwMS (*p* = 0.44). Mean Borg RPE scores after exercise were 10.6 ± 2.2 (falling within “fairly light” exercise category) in HP and 13.4 ± 1.8 (falling within the “somewhat hard” exercise category) in PwMS (*p* = 0.32).

### 3.2. IPS Results

A detailed description of the IPS results is provided in Table 1. Mean baseline SDMT-a raw scores were higher in HP than in PwMS (*p* < 0.05). The same directional difference was observed when SDMT-b was first administered (*p* < 0.05). Cycling-related SDMT change was 7.0 ± 6.4 points in HP and 1.9 ± 4.5 points in PwMS, corresponding to Mann–Whitney effect size of *r* = 0.49 (95% CI 0.05–0.76; *p* = 0.03). Comparisons following the other physiologic challenges did not meet the conventional *p* < 0.05 threshold.

Baseline SDMT-a raw scores were not correlated with improvement in consecutive SDMT scores in either group (*p* > 0.20). Baseline SDMT raw scores were not correlated with age, sex, ASP scores, or any clinical disease activity variable in PwMS (*p* > 0.20 for all comparisons).

### 3.3. Hemodynamic Results

**Figure 3:**
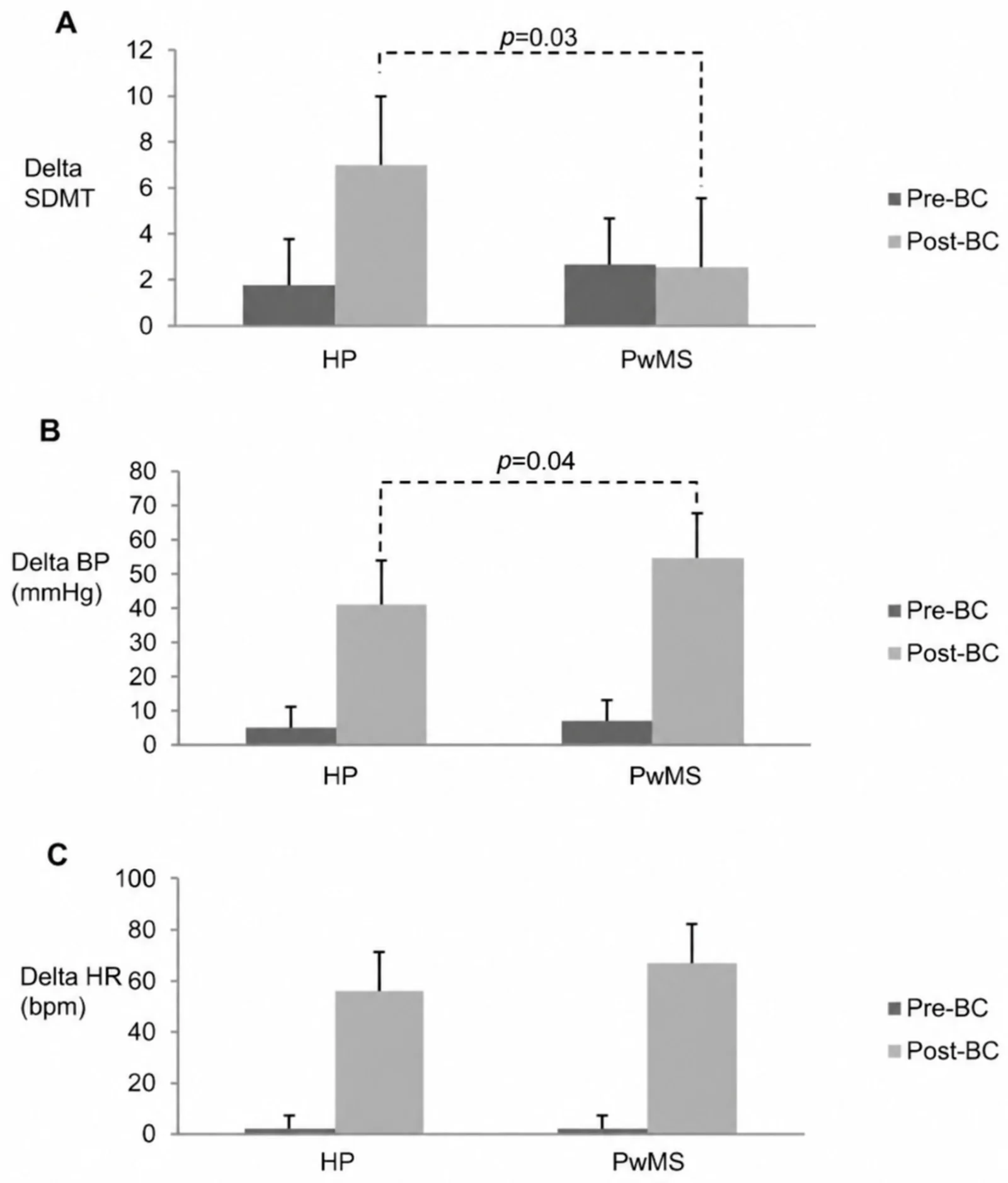
Comparison of changes in IPS, BP, and HR before and after cycling exercise in HP and PwMS. (**A**) Change in SDMT raw scores, (**B**) change in BP, and (**C**) change in HR before and after cycling exercise were compared between HP and PwMS. Between-group comparisons were performed using the Mann–Whitney U test. BC, cycling session; BP, blood pressure; HR, heart rate; PwMS, people with multiple sclerosis; SDMT, Symbol Digit Modalities Test.

A detailed description of heart rate and blood pressure values and their respective changes is provided in Table 1 and Table 2 and Figure 3. Mean systolic blood pressure during cycling was higher in PwMS than in HP (*p* < 0.05). The cycling-related increase in systolic blood pressure was 41.9 ± 27.2 mmHg in HP and 54.2 ± 17.2 mmHg in PwMS (*r* = 0.46 95% CI 0.02–0.75; *p* = 0.04). The remaining hemodynamic comparisons were not statistically significant; given the small sample size, these results should not be interpreted as demonstrating equivalence between groups. Heart rate and blood pressure changes during head tilt, isometric handgrip, and cycling were not correlated with each other.

Body temperatures before and after exercise were 36.7 °C and 36.6 °C in HP, and 37.0 °C and 36.9 °C in PwMS. Maximal handgrip pressure was 42.2 ± 5.6 Kg (mean ± SD) in HP and 29.8 ± 5.3 Kg (mean ± SD) in PwMS (*p* = 0.10). There were no significant associations between hemodynamic variables and age, sex, ASP scores, or any clinical disease activity variable in PwMS (*p* > 0.20 for all comparisons).

### 3.4. IPS/Hemodynamic Comparison

**Figure 4:**
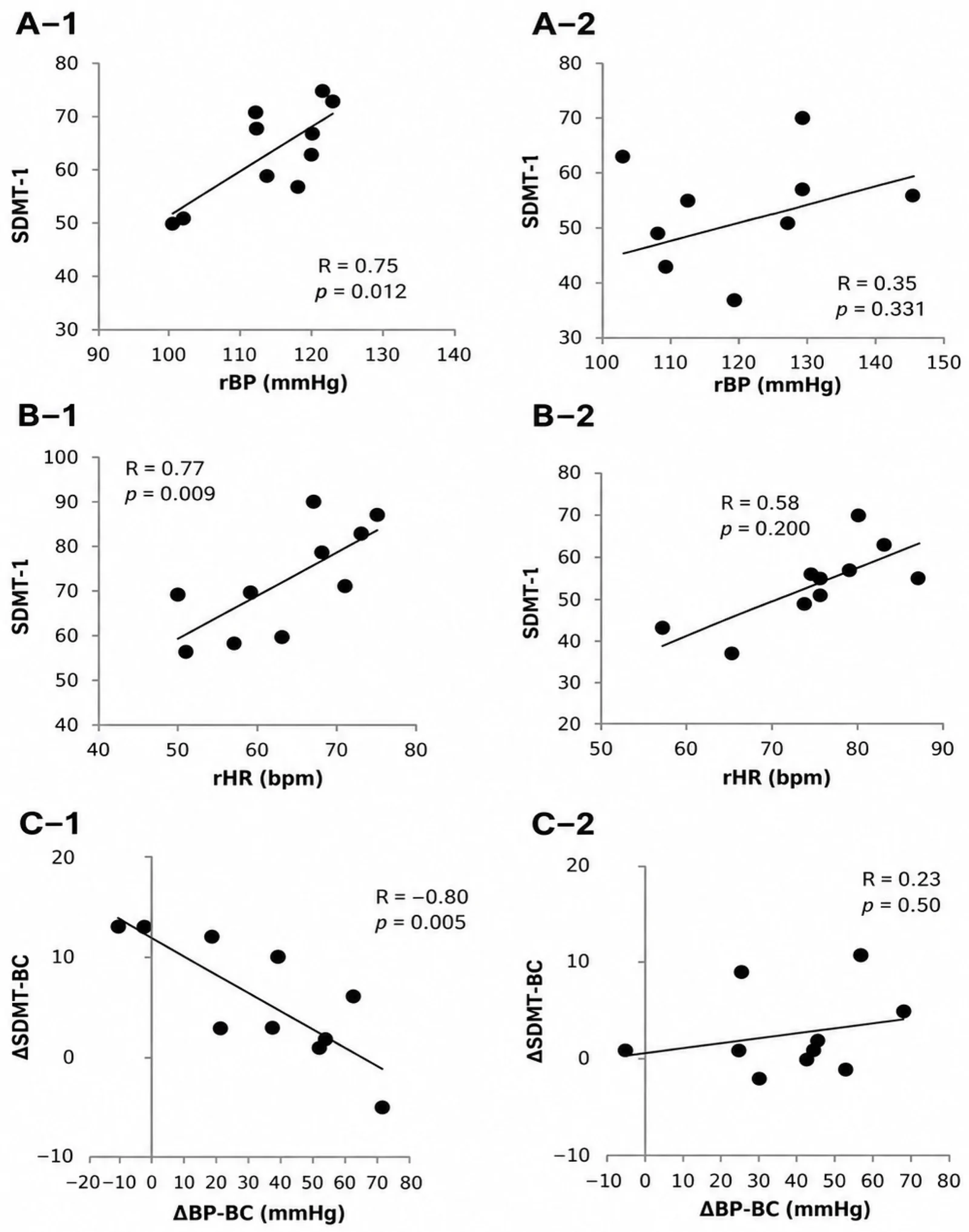
Correlations between IPS and hemodynamic measures in HP and PwMS. Correlations between SDMT raw scores and resting blood pressure are shown for (**A–1**) HP and (**A–2**) PwMS. Correlations between SDMT raw scores and resting heart rate are shown for (**B–1**) HP and (**B–2**) PwMS. Correlations between change in SDMT raw score and change in blood pressure during the cycling session are shown for (**C–1**) HP and (**C–2**) PwMS. Since two patients have very similar values, 2 dots overlap in (**A–2**). Correlations were evaluated using Spearman’s rank correlation coefficient. Bpm, beats per minute; mmHg, millimeters of mercury; PwMS, people with multiple sclerosis; R, rho; rBP, resting systolic blood pressure; rHR, resting heart rate; SDMT, Symbol Digit Modalities Test; ΔBP-BC, delta systolic blood pressure during the cycling session; ΔSDMT-BC, change in Symbol Digit Modalities Test score after the cycling session.

In HP, baseline SDMT-1 performance was positively correlated with resting systolic blood pressure (ρ = 0.75, 95% CI = 0.23–0.94; *p* = 0.012) and resting heart rate (ρ = 0.77, 95% CI = 0.27–0.94; *p* = 0.009). In PwMS, the corresponding estimates were ρ = 0.35, 95% CI = −0.36–0.80; *p* = 0.331 for resting systolic blood pressure and ρ = 0.58, 95% CI = −0.08–0.89; *p* = 0.2 for resting heart rate. Cycling-related SDMT change was inversely correlated with the cycling-related systolic blood pressure response in HP (ρ = −0.80, 95% CI = −0.95–−0.34; *p* = 0.005), whereas the corresponding estimate in PwMS was ρ = 0.23, 95% CI = −0.47–0.75; *p* = 0.5. SDMT change after handgrip did not show clear evidence of association with heart rate or blood pressure changes in either group (Figure 4).

## 4. Discussion

The present pilot study examined IPS and cardiovascular responses in people with RRMS compared with age- and sex-matched HP during several acute physiologic challenges. PwMS had lower baseline SDMT performance than HP, and a between-group effect was observed for cycling-related SDMT change (*r* = 0.49, 95% CI 0.05–0.76; *p* = 0.03). In addition, blood pressure during cycling and the blood pressure response to cycling were greater in PwMS despite a comparable TRIMP-standardized exercise workload. Relationships between cardiovascular measures and SDMT performance were observed in HP but not in PwMS. Together, these findings suggest that PwMS with mild disability may demonstrate both reduced IPS and differences in associations between cardiovascular responses and cognitive performance during acute physiologic stress, although the exploratory nature of this pilot study and potential practice effects warrant cautious interpretation. Lower baseline SDMT performance in PwMS is consistent with the established literature showing that IPS is among the cognitive domains most commonly and sensitively affected in MS [1,2,3,4]. In our study, reduced IPS was present despite mild neurologic disability and low relapse activity, and SDMT performance was not associated with EDSS, age at onset, annualized relapse rate, or time since the last relapse. These findings are in agreement with prior studies suggesting that cognitive dysfunction in MS may be partly dissociated from conventional physical disability measures and may occur even in ambulatory or mildly disabled individuals [1,22].

The most distinctive finding was that the change in SDMT performance differed between groups after cycling exercise. Although this finding must be interpreted in the context of repeated SDMT administration and potential practice effects, it suggests the possibility that dynamic aerobic exercise may reveal differences in cognitive–physiologic regulation that are not apparent during other forms of physiologic stress. Prior studies examining acute exercise and cognition in MS have yielded mixed results. Sandroff and colleagues reported improvements in aspects of cognitive performance after walking, cycling, and yoga, with later work in patients with RRMS suggesting benefits of treadmill exercise for cognitive inhibitory control [10,23]. Conversely, Lefferts and colleagues observed exercise-induced cardiovascular and cerebrovascular changes without a specific cognitive benefit compared with quiet rest [11]. Thus, our findings fit within a mixed literature and suggest that the SDMT response to acute aerobic exercise may vary between PwMS and HP, even in the absence of a significant within-group change in PwMS. Although cycling workload was standardized using TRIMP and did not differ between groups, PwMS had higher blood pressure during cycling and a greater exercise-induced increase in blood pressure. Thus, a comparable relative workload may impose a greater hemodynamic challenge in MS. Perceived exertion (Borg RPE) ratings were also numerically higher in the MS group, although this difference was not statistically significant. An alternative explanation is that baseline aerobic fitness, which was not directly measured, differed between groups, given that aerobic fitness is often reduced in individuals with MS and may affect cardiovascular responses to exercise [24].

These findings are compatible with previous evidence that cardiovascular autonomic dysfunction occurs in MS, with prevalence estimates varying according to the criteria and tests used [25,26]. Our previous meta-analysis estimated the prevalence of autonomic dysfunction to range from approximately 19% to 42% in PwMS, depending on whether one or two abnormal cardiovascular autonomic tests were required [26]. However, the present results should not be interpreted as evidence of generalized autonomic failure, because heart rate and blood pressure responses were otherwise largely comparable between groups. The lack of correlation among responses to different physiologic challenges further suggests that these maneuvers reflect distinct physiologic processes. Head-up tilt primarily challenges orthostatic regulation, handgrip elicits sympathetic activation and a pressor response during sustained static contraction, whereas cycling combines increased cardiac output, metabolic demand, skeletal muscle activity, and vascular regulation. The lack of a handgrip–SDMT association further suggests that blood pressure elevation alone does not explain the cognitive findings, because cycling engages broader metabolic, respiratory, cardiovascular, and cerebrovascular responses than static handgrip [27]. Similarly, the absence of broad orthostatic abnormalities may reflect the small sample size, mild disability, exclusion of participants with major autonomic or cardiovascular disorders, or the limited duration or intensity of the tilt challenge [28].

A particularly novel finding, although still exploratory, was the difference in cardiovascular–cognitive associations between groups. In HP, baseline SDMT performance was associated with resting heart rate and blood pressure, whereas cycling-related SDMT change was negatively associated with the blood pressure response. These relationships were absent in PwMS, suggesting, but not confirming, a possible disruption of the normal association between cardiovascular state and IPS. In HP, cardiovascular arousal may influence cognition through alertness, catecholaminergic activation, cerebral perfusion, and neurovascular coupling. Meta-analyses indicate that acute exercise has small effects on cognition, and that processing-speed outcomes may be sensitive to exercise intensity and physiologic arousal [29,30]. In MS, lesion burden and distributed network disruption [1,5], fatigue [31], autonomic dysfunction [25], and vascular dysregulation [8] may alter these effects.

This interpretation is consistent with evidence that vascular and cerebrovascular abnormalities contribute to cognitive dysfunction in MS. Lower cerebral arterial flow, altered perfusion, vascular comorbidity, and reduced arterial compliance have been associated with poorer cognitive performance [6,7,8,9]. Cerebral hypoperfusion has also been proposed as a potential contributor to chronic hypoxia, axonal injury, fatigue, and cognitive impairment [32]. Nevertheless, cerebral blood flow abnormalities are not universal in MS. The ROCHIMS trial did not identify reduced cerebral blood flow in mildly affected RRMS and suggested that cerebral hypoperfusion may depend on disease activity or patient subgroup [33]. This heterogeneity is relevant to the present mildly disabled sample, particularly because cerebral blood flow was not measured directly.

Practice effects must be considered when interpreting repeated SDMT assessments, particularly when tests are administered over short intervals [34]. Two SDMT versions were used to minimize repeated exposure to the same test, and the same version was consistently used within paired comparisons to prevent confounding physiologic effects with test version differences. However, this approach led to unequal exposure to the two SDMT versions throughout the protocol. Moreover, because the intervention sequence was fixed, cycling occurred after all preceding orthostatic and handgrip interventions and was evaluated using SMDT-a at both SDMT-7 and SDMT-8. Therefore, cumulative practice effects and increased familiarity with SDMT-a may have influenced the observed cycling-related change and cannot be ruled out as alternative explanations for the between-group difference. Although baseline SDMT performance did not correlate with subsequent change, the possibility of learning effects due to repeated administration remains. Future research should incorporate additional alternate SDMT forms, counterbalance test versions and intervention order, or include a repeated-testing control condition to more effectively differentiate physiologic effects from practice effects. Body temperature did not increase after cycling in either group, making heat-related neurologic worsening an unlikely explanation for the post-exercise SDMT findings. These findings suggest that the intervention acted primarily as a cardiovascular and metabolic challenge rather than a thermal stressor, although future studies should include more direct measures of fatigue, perceived exertion, autonomic function, and cerebrovascular physiology.

The main limitation of this pilot study was the small sample size of 10 participants per group, which reduced statistical power and increased the possibility of type I and type II errors. Because analyses were exploratory and were not corrected for multiple comparisons, significant group differences and correlations should be considered hypothesis-generating. The MS cohort had heterogeneity in DMT exposure, and one participant was receiving multiple concomitant medications with potential hemodynamic effects. Although medications with possible interference were withheld for 24 h prior to testing, the possibility of chronic or residual effects cannot be excluded. Given the small sample size, it was not possible to assess or adjust for the potential effects of DMT type, treatment duration, or concomitant medications on cardiovascular responses or cognitive performance. Therefore, treatment-related effects remain potential confounders, particularly in the interpretation of cardiovascular findings. Using a fixed intervention sequence and repeated SDMT administration also limits the ability to distinguish intervention-specific effects from potential practice and order effects, especially in the context of the cycling assessment. Unmeasured factors such as cerebral blood flow, arterial stiffness, heart rate variability, baroreflex sensitivity, fitness, fatigue, depression, and sleep may also have influenced the results. Larger studies incorporating direct autonomic and cerebrovascular measures, including heart rate variability, baroreflex sensitivity, transcranial Doppler or arterial spin-labeling MRI, cerebral oxygenation, and arterial stiffness, as well as counterbalanced intervention sequences and SDMT versions will be needed to clarify the mechanisms underlying altered cardiovascular–cognitive relationships in MS.

## 5. Conclusions

In conclusion, this pilot study found lower IPS in RRMS and an exploratory between-group effect in cycling-related SDMT change. PwMS also had a greater cycling-related systolic blood pressure response. The observed correlation patterns raise the possibility of differences in cardiovascular–cognitive relationships between PwMS and HP, although wide confidence intervals preclude firm conclusions. These findings should be considered hypothesis-generating and require confirmation in larger mechanistic studies incorporating direct autonomic and cerebrovascular measures.

## Figures and Tables

**Table 1:** Information processing speed, systolic blood pressure and heart rate values of HP and PwMS.

	HP			PwMS		
	SDMT Raw Scores and Change ± SD	SBP Values (mmHg) ± SD	HR Values (bpm) ± SD	SDMT Raw Scores and Change ± SD	SBP Values (mmHg) ± SD	HR Values (bpm) ± SD
**SDMT-1**	62.3 ± 8.7 *	**113.1 ± 8.9**	**71.2 ± 11.3**	49.3 ± 12.8 *	**120.9 ± 12**	**74.1 ± 8.1**
SDMT-1 to 3	6.2 ± 4.7	113.3 ± 7.7	68.1 ± 11.6	4.8 ± 6.8	119.0 ± 12.1	72.1 ± 7.8
SDMT-3 to 5	2.2 ± 5.3	113.3 ± 9.9	66.9 ± 12.2	4.3 ± 2.0	115.0 ± 12.1	70.7 ± 9.0
SDMT-5 to 7	1.5 ± 3.1	156.9 ± 8.6	90.6 ± 10.6	2.7 ± 2.4	156.2 ± 13.5	85.2 ± 13.2
SDMT-7 to 8	7.0 ± 6.4 *	154.4 ± 20.3 *	122.4 ± 9.6	1.9 ± 4.5 *	169.7 ± 25.7 *	137.2 ± 23.8
**SDMT-2**	58.9 ± 8.6 *	**113.2 ± 7.2**	**69.6 ± 11.7**	47.8 ± 11.2 *	**119.0 ± 12.1**	**72.1 ± 7.8**
SDMT-2 to 4	2.8 ± 3.7	108.5 ± 6.9	82.2 ± 15.1	3.1 ± 3.5	117.1 ± 12.0	74.4 ± 8.5
SDMT-4 to 6	3.7 ± 5.1	109.9 ± 9.0	76.3 ± 13.5	4.9 ± 4.7	109.9 ± 9.0	78.7 ± 8.5

Raw scores for the two versions of SDMT used and changes on the consecutive SDMT administration within the same version (SDMT-a: 1, 3, 5, 7, and 8; SDMT-b: 2, 4, and 6). The BP and HR averages at the time of each SDMT administration are presented. bpm, beats per minute; HR, heart rate; mmHg, millimeters of mercury; PwMS, people with multiple sclerosis; SBP, systolic blood pressure; SD, standard deviation; SDMT, Symbol Digit Modalities Test. * Represents groups with significant difference (*p* < 0.05).

**Table 2:** BP and HR changes during HUT, HG and BC in HP and PwMS.

	HP		PwMS	
	ΔBP (mmHg)	ΔHR (bpm)	ΔBP (mmHg)	ΔHR (bpm)
Change during HUT (mean ± SD)	−5.5 ± 8.8	19.0 ± 11.0	0.1 ± 14.3	15.8 ± 6.2
Change during HG (mean ± SD)	41.7 ± 11.1	23.6 ± 4.1	32.4 ± 14.7	16.8 ± 8.2
Change during BC (mean ± SD)	41.9 ± 27.2 *	48.3 ± 20.1	54.2 ± 17.2 *	61.6 ± 20.6

BC, cycling session; BP, blood pressure; bpm, beats per minute; HG, handgrip; HR, heart rate; HUT, head-up tilt; mmHg, millimeters of mercury; PwMS, people with multiple sclerosis; SD, standard deviation. * Represents group with significant difference (*p* < 0.05).

## Data Availability

The data presented in this study is available on request from the corresponding author.

## Abbreviations

The following abbreviations are used in this manuscript:

ASP	Autonomic Symptom Profile
EDSS	Expanded Disability Status Scale
IPS	Information Processing Speed
HP	Healthy Participant
mmHg	Millimeters of mercury
MS	Multiple Sclerosis
PwMS	People with MS
rBP	Resting Blood Pressure
rHR	Resting Heart Rate
RPE	Rating of Perceived Exertion
RRMS	Relapsing-Remitting MS
SD	Standard Deviation
SDMT	Symbol Digit Modalities Test
TRIMP	Training Impulse
ΔBP-BC	Delta Systolic Blood Pressure during the Cycling session
ΔBP-HDT	Delta Systolic Blood Pressure during the Head-Down Tilt
ΔBP-HG	Delta Systolic Blood Pressure during the Handgrip
ΔBP-HUT	Delta Systolic Blood Pressure during the Head-Up Tilt
ΔHR-BC	Delta Heart Rate during the Cycling session
ΔHR-HDT	Delta Heart Rate during the Head-Down Tilt
ΔHR-HG	Delta Heart Rate during the Handgrip
ΔHR-HUT	Delta Heart Rate during the Head-Up Tilt
